# Protein Fragmentation As a Regulatory Mechanism: Insights from Two Different Ca^2+^ Channels, RyR1 and IP_3_R

**DOI:** 10.3389/fphys.2016.00655

**Published:** 2017-01-04

**Authors:** Daria Neyroud

**Affiliations:** Faculty of Biology and Medicine, Institute of Sport Sciences, University of LausanneLausanne, Switzerland

**Keywords:** protein fragmentation, ryanodine receptor 1, inositol 1, 4, 5-triphosphate receptor, protein regulation, Ca^2+^ handling

The ryanodine receptor 1 (RyR1) is the major skeletal muscle Ca^2+^ release channel and as such a key player in excitation-contraction coupling. Intriguingly, a recent paper reported RyR1 fragmentation 24 h after high-intensity interval exercise (six 30-s all-out cycling bouts with 4 min recoveries in between) in *vastus lateralis* muscle biopsies taken from recreationally active men (Place et al., 2015). In contrast to what could have been hypothesized based on the role of RyR1 in excitation-contraction coupling, this RyR1 fragmentation did not appear to result in excitation-contraction coupling failure, as both maximal voluntary contraction force and forces evoked by supramaximal electrical stimulations had fully recovered 24 h after exercise. Overall, these results suggest that fragmented RyR1 retain their capacity to release Ca^2+^ in response to an action potential.

If at first this result might appear surprising, as fragmentation is generally thought to result in dysfunctional channels, a recent review published by Yule and colleagues highlighted that fragmentation might actually serve as a regulating mechanism, at least for the inositol 1,4,5-triphosphate receptor (IP_3_R), the major Ca^2+^ release channel in non-excitable cells (Wang et al., 2016). Briefly, in addition of being physiologically activated by inositol 1,4,5-triphosphate (IP_3_), IP_3_R can be modulated by intracellular Ca^2+^, ATP, cAMP, as well as by post-translational changes such as phosphorylation and redox modifications, similarly to RyR1 (Lanner et al., 2010). By proteolytic cleavage, also IP_3_R may become fragmented (Hirota et al., 1999; Kopil et al., 2011). Early results suggested dysfunctional leaky fragmented IP_3_Rs (Assefa et al., 2004; Verbert et al., 2008; Kopil et al., 2011), but Wang et al. (2016) then argued that the model used in the earlier studies presented important limitations precluding such conclusions. For example in the study of Assefa et al. (2004), a construct encoding only the IP_3_R caspase-cleaved C-terminal domain was expressed in DT40-3KO cells (chicken B-lymphocytes with all IP_3_R isoforms knocked-out) and resulted in an enhanced Ca^2+^ leak. According to Wang et al. (2016) these results are not informative of the functionality of fragmented IP_3_Rs as (i) the C-terminal portion of the IP_3_R might have been overexpressed, and (ii) it was expressed in a background without the IP_3_R N-terminal cytoplasmic domain. Using DT40-3KO cells expressing IP_3_R isoform 1 (IP_3_R1), they then showed, by separation on a native non-denaturating gel, that both the N- and C-terminal fragments of the channel remained associated following IP_3_R fragmentation induced by staurosporine (Alzayady et al., 2013). To further ensure that the observed result was not caused by full-length IP_3_R remaining after possibly incomplete staurosporine treatment, they used an approach in the absence of full-length IP_3_R1 and constructed dual promoter vectors encoding complementary N- and C-terminal domains (Alzayady et al., 2013). Intriguingly, they found, both by co-immunoprecipitation and native gel separation, that the complementary IP_3_R1 fragments assembled into tetrameric IP_3_R1. They further demonstrated that these assembled N- and C- complementary fragments did not lead to increased basal cytosolic [Ca^2+^] ([Ca^2+^]_i_), nor did it cause endoplasmic reticulum store depletion, as expected by the leaky channel hypothesis. Those authors further showed that IP_3_R1 assembled from C- and N- complementary fragments could still be regulated by IP_3_, suggesting conserved functionality.

Turning back to the ryanodine receptors (RyR), which have a similar domain structure as IP_3_Rs, it was shown that overexpression of the ryanodine receptor type 2 (RyR2) C-terminal domain resulted in a leaky channel, whereas co-expression of both N- and C-terminal domains restored normal RyR2 function (George et al., 2004). It therefore appears that both IP_3_Rs and RyRs might still be functional when fragmented. Yet, when the functional consequences of RyR1 fragmentation were investigated by mimicking the short term high-intensity interval exercise as used in the human experiments, by electrically stimulating intact mouse *flexor digitorum brevis* single fibers, reduced tetanic and increased baseline [Ca^2+^]_i_ were observed 3 h after the intense stimulation, when RyR1 was fragmented, indicative of a sarcoplasmic reticulum Ca^2+^ leak (Place et al., 2015). Although at first glance it might thus appear that fragmentation affected the IP_3_R and RyR1 differently in terms of its effect on Ca^2+^ handling (i.e., fragmented IP_3_R was reported as non-leaky and RyR1 as leaky), it is important to mention that the Ca^2+^ leak detected when the RyR1 was fragmented was of a very low magnitude ([Ca^2+^]_i_ was increased by ~20 nM, Figure 4E in Place et al., 2015), possibly below detection levels for lymphocyte cells. It can therefore be suggested that low level Ca^2+^ leak, resulting from “functional” fragmentation of RyR1, might play a role in physiological adaptation (“good leak”) as opposed to a large and sustained Ca^2+^ leak leading to defective excitation-contraction coupling and ultimately cell death (“bad leak”).

In conclusion, even if protein fragmentation is often considered part of the catabolic pathway, fragmentation might not necessarily lead to non-functional channels. Rather, proteolytic fragmentation of an ion channel might modulate its function and regulate downstream cellular pathways in a beneficial manner. If this novel potential role of fragmentation as a possible mechanism of channel regulation allows explaining the absence of excitation-contraction coupling failure when RyR1 is fragmented, it warrants further research to better understand its importance and consequences in cellular events.

## Author contributions

DN drafted the manuscript and approved the final version.

### Conflict of interest statement

The author declares that the research was conducted in the absence of any commercial or financial relationships that could be construed as a potential conflict of interest.

## References

[B1] Alzayady K. J. Chandrasekhar R. Yule D. I. (2013). Fragmented inositol 1,4,5-trisphosphate receptors retain tetrameric architecture and form functional Ca^2+^ release channels. J. Biol. Chem. 288, 11122–11134. 10.1074/jbc.M113.453241 23479737 PMC3630841

[B2] Assefa Z. Bultynck G. Szlufcik K. Nadif Kasri N. Vermassen E. Goris J.. (2004). Caspase-3-induced truncation of type 1 inositol trisphosphate receptor accelerates apoptotic cell death and induces inositol trisphosphate-independent calcium release during apoptosis. J. Biol. Chem. 279, 43227–43236. 10.1074/jbc.M403872200 15284241

[B3] George C. H. Jundi H. Thomas N. L. Scoote M. Walters N. Williams A. J.. (2004). Ryanodine receptor regulation by intramolecular interaction between cytoplasmic and transmembrane domains. Mol. Biol. Cell 15, 2627–2638. 10.1091/mbc.E03-09-0688 15047862 PMC420088

[B4] Hirota J. Furuichi T. Mikoshiba K. (1999). Inositol 1,4,5-trisphosphate receptor type 1 is a substrate for caspase-3 and is cleaved during apoptosis in a caspase-3-dependent manner. J. Biol. Chem. 274, 34433–34437. 10.1074/jbc.274.48.34433 10567423

[B5] Kopil C. M. Vais H. Cheung K. H. Siebert A. P. Mak D. O. Foskett J. K.. (2011). Calpain-cleaved type 1 inositol 1,4,5-trisphosphate receptor (InsP(3)R1) has InsP(3)-independent gating and disrupts intracellular Ca(2^+^) homeostasis. J. Biol. Chem. 286, 35998–36010. 10.1074/jbc.M111.254177 21859719 PMC3195633

[B6] Lanner J. T. Georgiou D. K. Joshi A. D. Hamilton S. L. (2010). Ryanodine receptors: structure, expression, molecular details, and function in calcium release. Cold Spring Harb. Perspect. Biol. 2:a003996. 10.1101/cshperspect.a003996 20961976 PMC2964179

[B7] Place N. Ivarsson N. Venckunas T. Neyroud D. Brazaitis M. Cheng A. J.. (2015). Ryanodine receptor fragmentation and sarcoplasmic reticulum Ca^2+^ leak after one session of high-intensity interval exercise. Proc. Natl. Acad. Sci. U.S.A. 112, 15492–15497. 10.1073/pnas.1507176112 26575622 PMC4687604

[B8] Verbert L. Lee B. Kocks S. L. Assefa Z. Parys J. B. Missiaen L.. (2008). Caspase-3-truncated type 1 inositol 1,4,5-trisphosphate receptor enhances intracellular Ca^2+^ leak and disturbs Ca2^+^ signalling. Biol. Cell 100, 39–49. 10.1042/BC20070086 17868032 PMC2909191

[B9] Wang L. Alzayady K. J. Yule D. I. (2016). Proteolytic fragmentation of inositol 1,4,5-trisphosphate receptors: a novel mechanism regulating channel activity? J. Physiol. (Lond). 594, 2867–2876. 10.1113/JP271140 26486785 PMC4887670

